# Supplementary material for: Developmental disruption of the mitochondrial fission gene drp-1 extends the longevity of daf-2 insulin/IGF-1 receptor mutant
Source: GeroScience. 2024 Jul 19;47(1):877–902. doi: 10.1007/s11357-024-01276-z (PMC11872967; doi:10.1007/s11357-024-01276-z)
Supplement: Supplementary file 1 — Supplementary file1 (PDF 3499 KB) [file 11357_2024_1276_MOESM1_ESM.pdf]

Supplemental Figures

for

Developmental disruption of the mitochondrial fission gene *drp-1* extends the longevity of *daf-2* insulin/IGF-1 receptor mutant

Annika Traa, Aura A. Tamez-González, Jeremy M. Van Raamsdonk

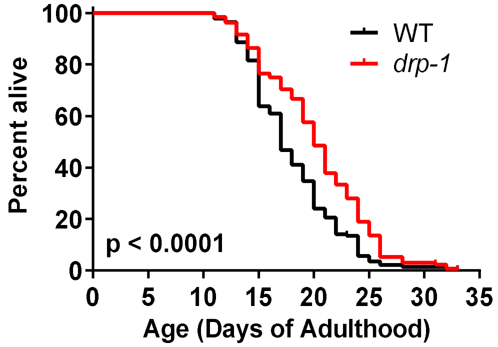

Fig. S1. Disruption of *drp-1* results in a small increase in lifespan. *drp-1* deletion mutants live longer than wild-type worms but the magnitude of the lifespan increase is small. Statistical significance was assessed using a log-rank test. WT N=141, *drp-1* N=132, temperature = 20°C.

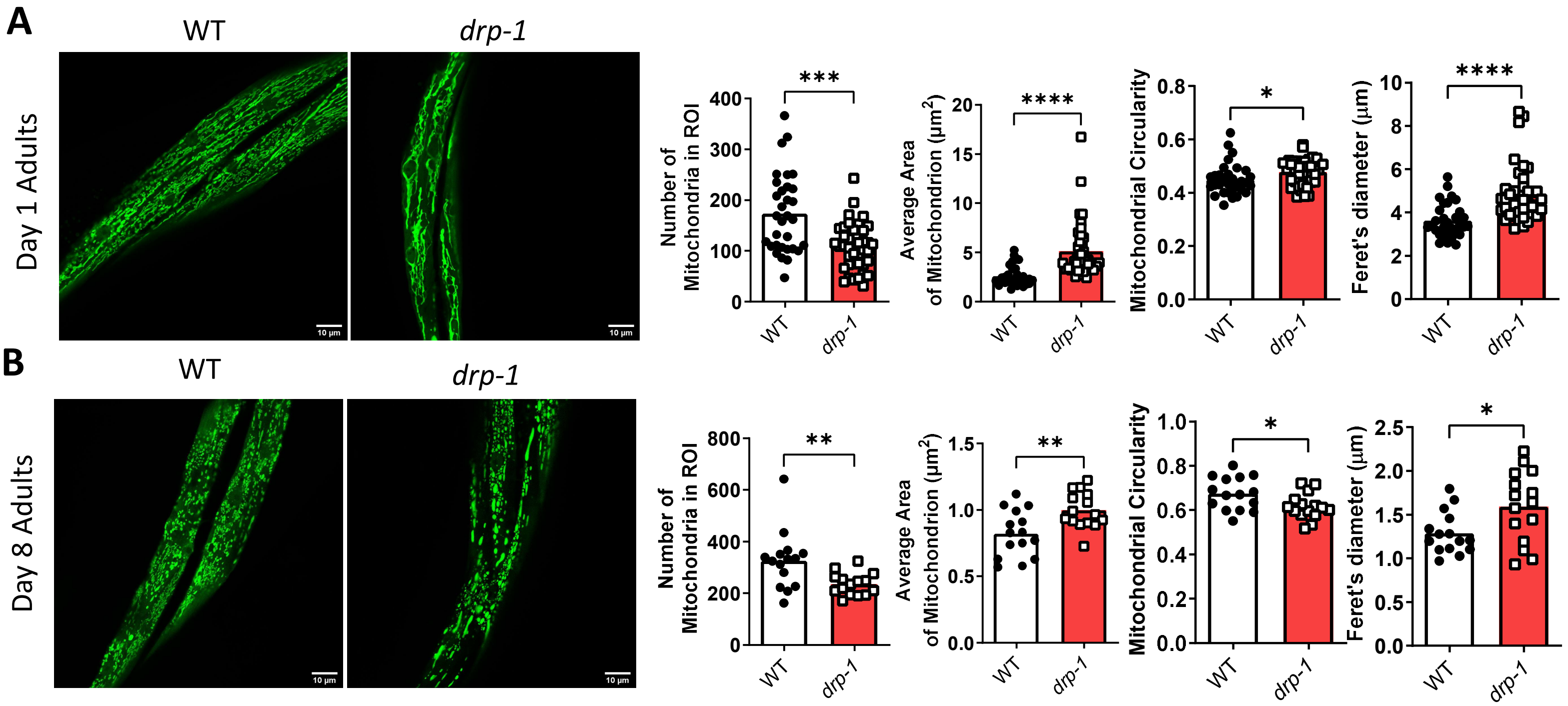

Fig. S2. Disruption of *drp-1* increases mitochondrial network connectivity in wild-type animals. At day 1 of adulthood, disruption of *drp-1* in wild-type animals decreases the number of mitochondria, increases average mitochondrial area, increases circularity and increases ferret's diameter (**A**). These measurements are similar at day 8, except that disruption of *drp-1* decreases circularity (**B**). Three biological replicates were imaged. Statistical significance was assessed using a student's t-test. Error bars indicate SEM. *$p<0.05$, **$p<0.01$, ***$p<0.001$, ****$p<0.0001$. Scale bar indicates 10 µm.

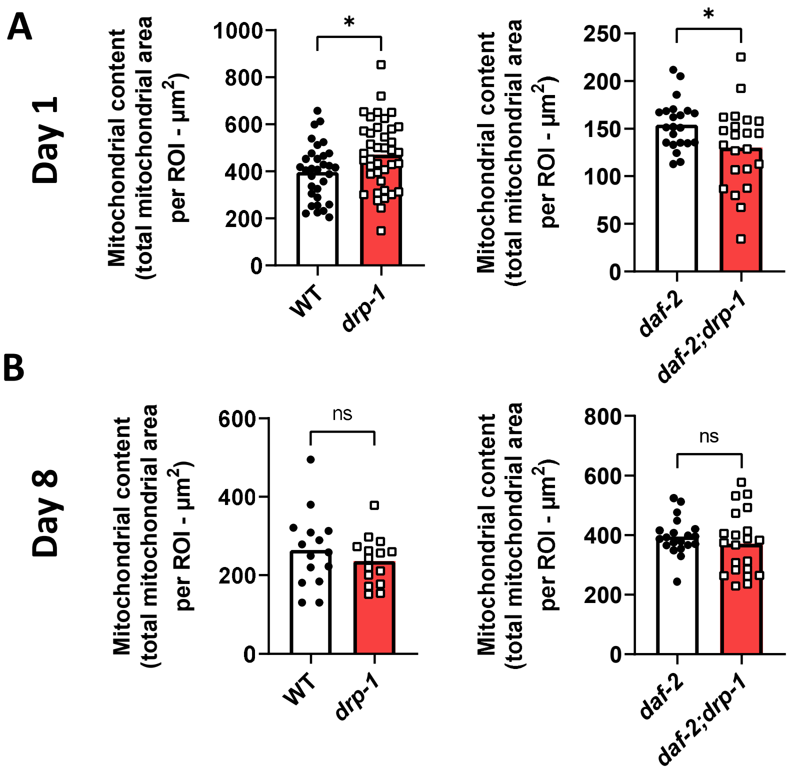

Fig. S3 . Mitochondrial content. At day 1 of adulthood, disruption of *drp-1* increases mitochondrial content in wild-type worms but decreases mitochondrial content in *daf-2* animals (**A**). The differences in mitochondrial content from the inhibition of *drp-1* are no longer present at day 8 of adulthood (**B**). Statistical significance was assessed using a student's t-test. Error bars indicate SEM. *p<0.05.

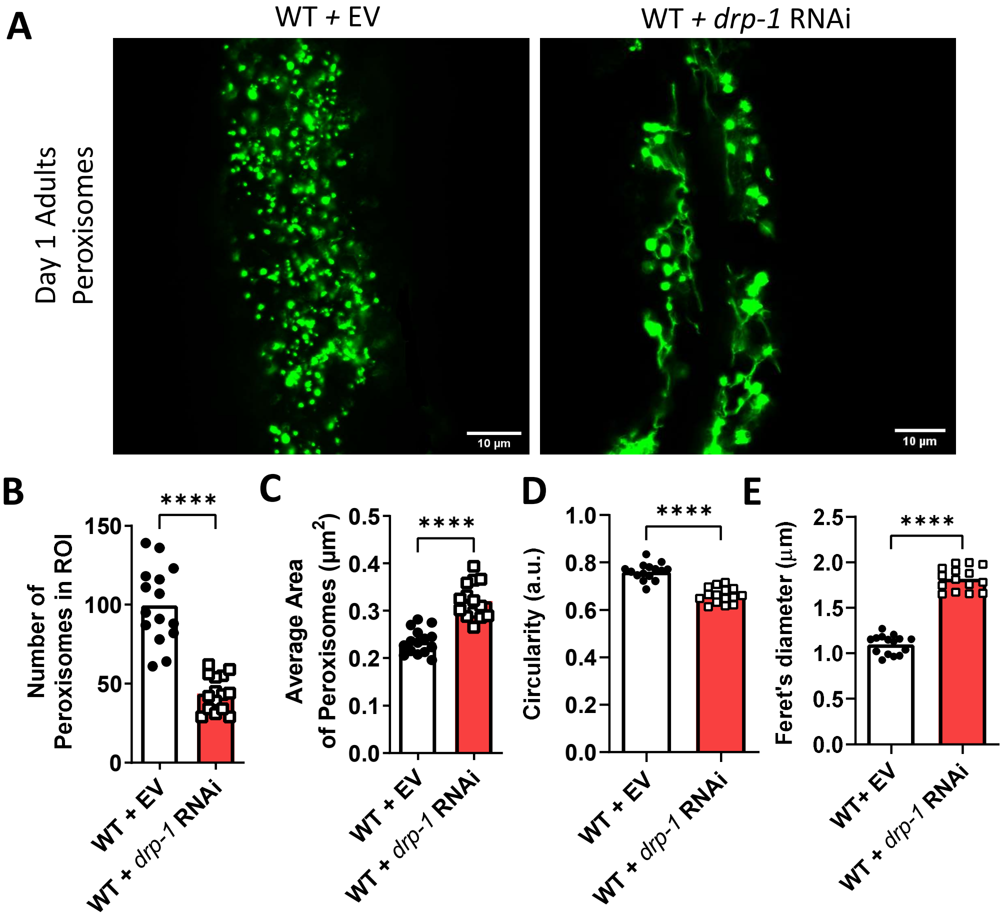

Fig. S4. Disruption of *drp-1* increases peroxisomal network formation. Treatment of wild-type worms with *drp-1* RNAi resulted in larger peroxisome networks with increased connectivity (**A**). Quantification of peroxisomal morphology demonstrated that *drp-1* RNAi decreased peroxisome numbers (**B**), increased peroxisome area (**C**), decreased peroxisome circularity (**D**) and increased the Feret's diameter of peroxisomes (**E**). All of these changes are consistent with decreasing peroxisomal fragmentation. Statistical significance was assessed using a student's t-test. Error bars indicate SEM. **** $p<0.0001$. Scale bar indicates 10 µm.

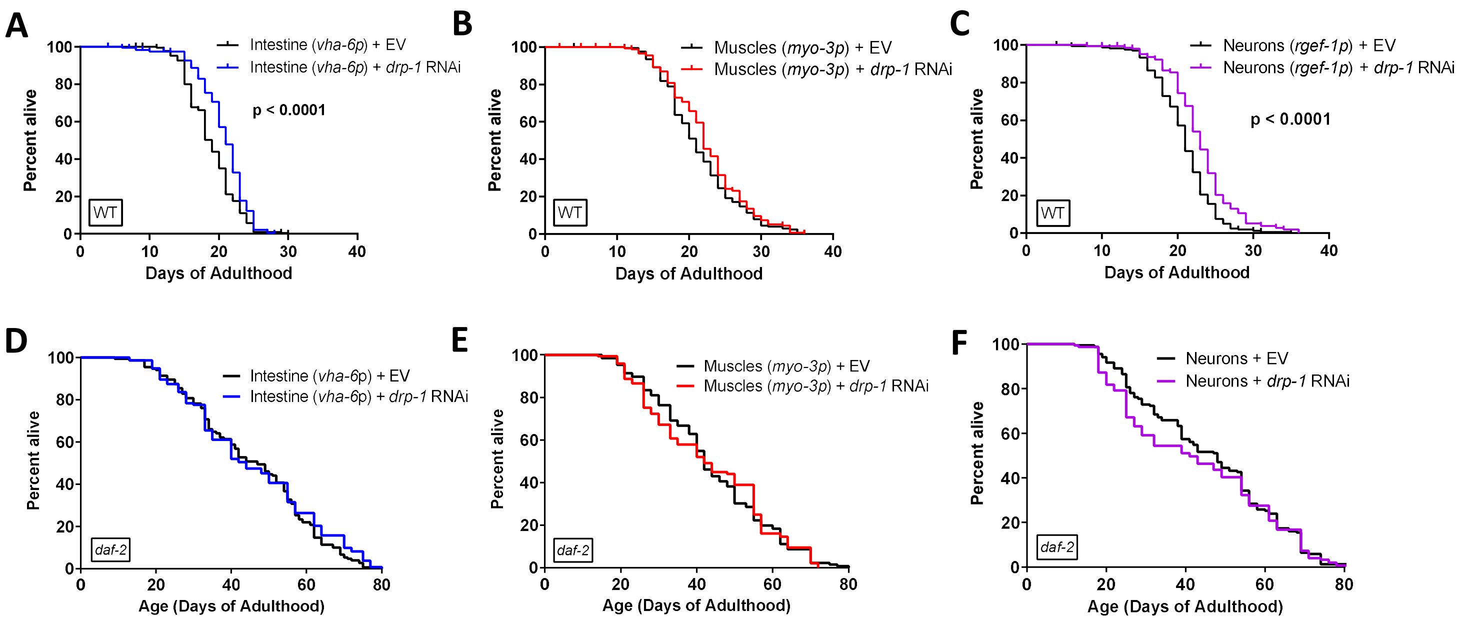

Fig. S5. Tissue-specific knockdown of *drp-1* does not extend *daf-2* lifespan. Compared to animals fed empty-vector (EV) control bacteria, wild-type animals with disruption of *drp-1* in the intestine had increased lifespan (**A**). Disruption of *drp-1* in the muscles of wild-type animals did not increase lifespan compared to animals fed EV (**B**). Disruption of *drp-1* in the neurons of wild-type animals increases lifespan compared to animals fed EV (**C**). In *daf-2* animals, disruption of *drp-1* in the intestine (**D**), muscles (**E**) or neurons (**F**) of *daf-2* animals does not affect lifespan compared to animals fed EV. Four biological replicates were performed. Sample size and raw lifespan data can be found in **Table S1**. Lifespan was performed at 20°C. Statistical significance was assessed using the log-rank test. *drp-1* RNAi data is from Fig. 4 where control is worms with a *sid-1* mutation treated with *drp-1* RNAi.

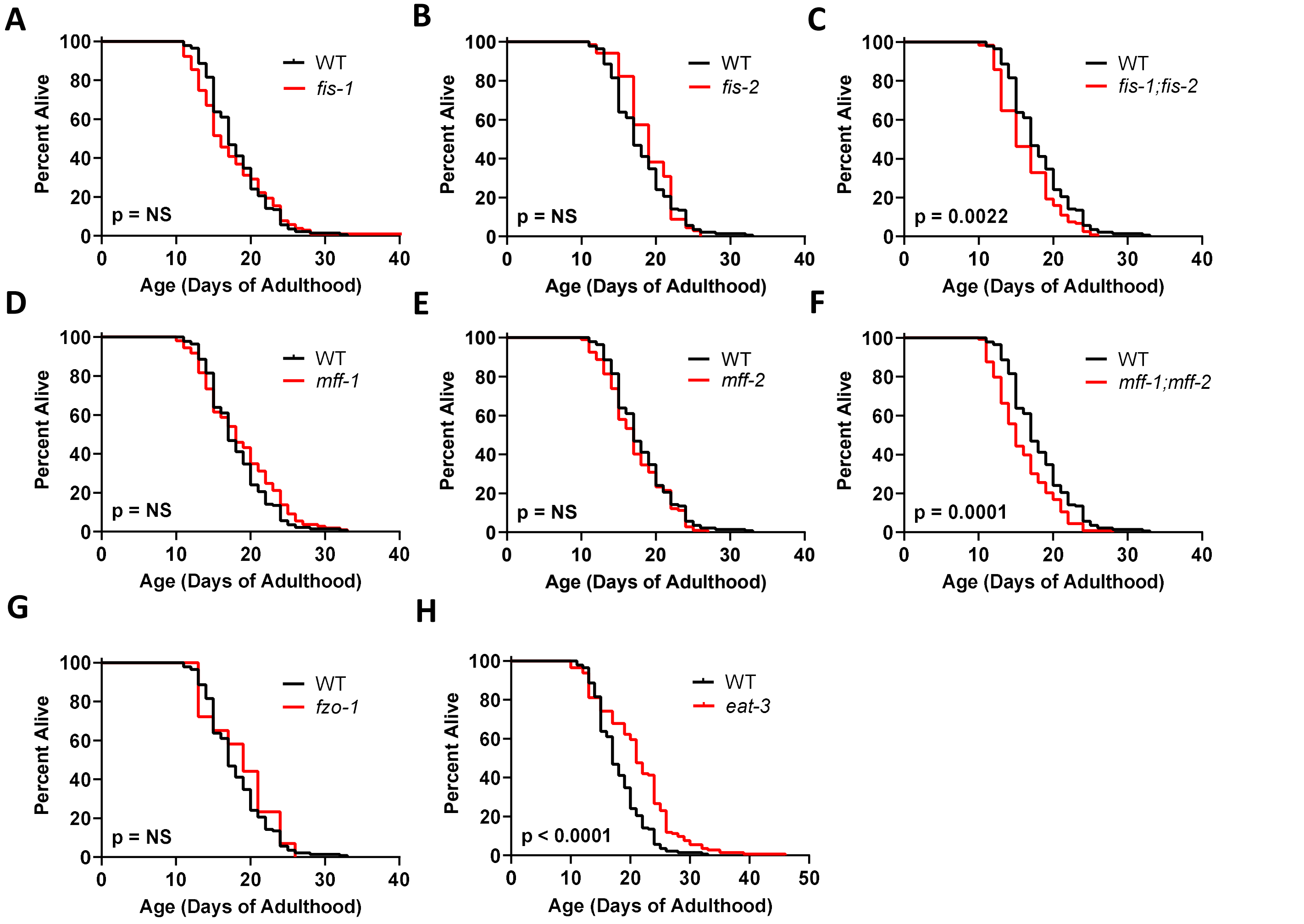

Fig. S6. Effect of disrupting mitochondrial fission or fusion genes on lifespan in wild-type worms. Disruption of either *fis-1* (**A**), or *fis-2* (**B**) individually did not affect wild-type lifespan, while *fis-1;fis-2* double mutants exhibited a slight decrease in longevity (**C**). Similarly, *mff-1* (**D**) and *mff-2* (**E**) single mutants have a normal lifespan, while *mff-1;mff-2* double mutants exhibit decreased longevity (**F**). While deletion of the mitochondrial fusion gene *fzo-1* did not affect lifespan (**G**), disruption of *eat-3* extended longevity (**H**). At least three biological replicates were performed. Sample size and raw lifespan data can be found in **Table S1**. Lifespan was performed at 20°C. Statistical significance was assessed using a log-rank test.

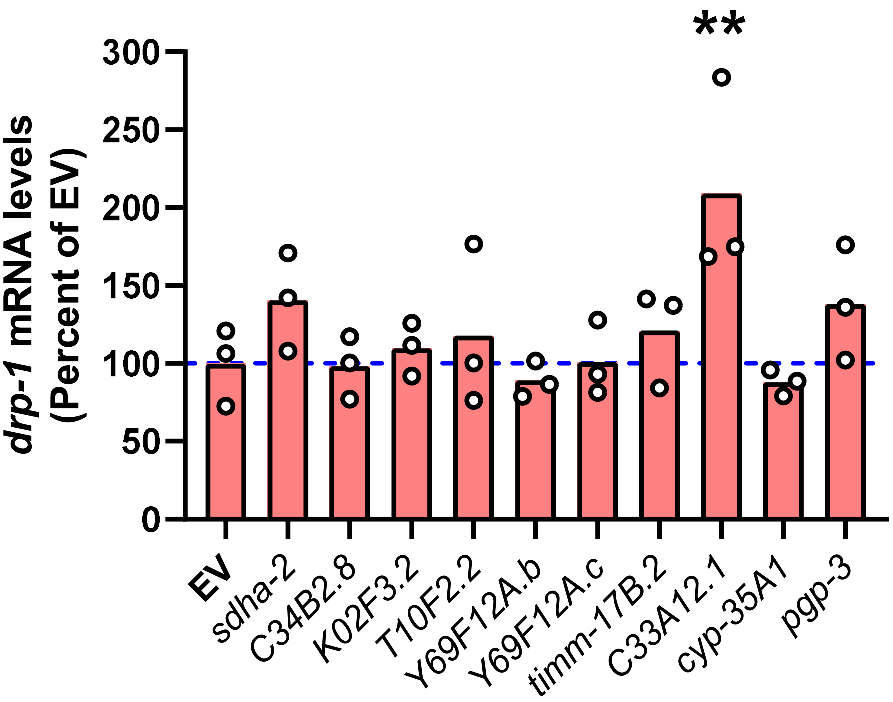

Fig. S7. RNAi clones that decrease mitochondrial fragmentation and increase lifespan do not decrease *drp-1* levels. Quantitative RT-PCR was used to measure *drp-1* mRNA levels after treatment with RNAi clones that were previously shown to decrease mitochondrial fragmentation and shown here to increase lifespan (**Fig. 6**). None of these RNAi clones decreased the expression of *drp-1.* This indicates that these RNAi clones decrease mitochondrial fragmentation and increase lifespan without affecting the levels of *drp-1*. Three biological replicates were performed. Statistical significance was assessed using a one-way ANOVA with Dunnett's multiple comparison test. ** $p<0.01$.

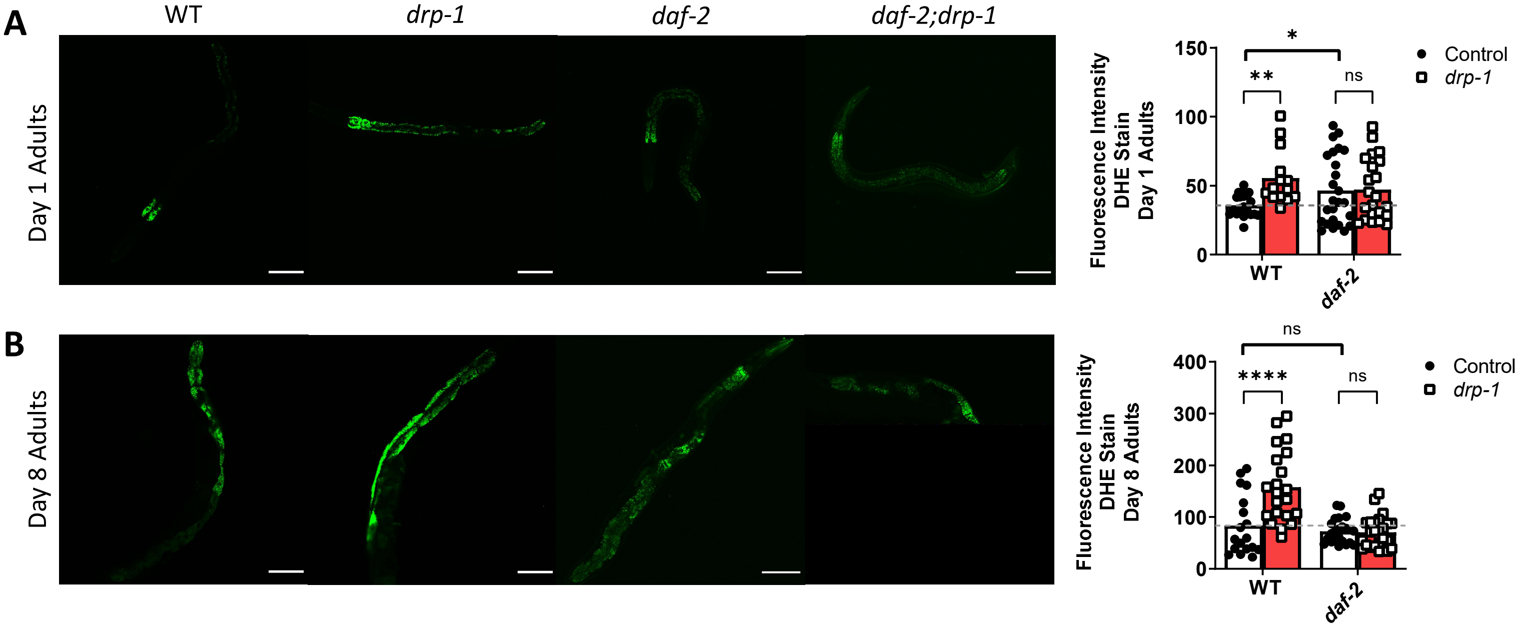

Fig. S8. Disruption of *drp-1* increases ROS levels in wild-type worms but not *daf-2* mutants. ROS levels indicated by whole-worm dihydroethidium (DHE) staining are higher in *drp-1* worms compared to wild-type worms at day 1 of adulthood, while *daf-2;drp-1* worms do not have increased ROS levels compared to *daf-2* worms (**A**). Similarly, at day 8 of adulthood, ROS levels remain higher in *drp-1* worms compared to wild-type, while *daf-2;drp-1* worms continue to show ROS levels similar to *daf-2* (**B**). Three biological replicates were performed. Statistical significance was assessed using a two-way ANOVA with Šidák's multiple comparisons test. Error bars indicate SEM. * $p<0.05$, ** $p<0.01$, **** $p<0.0001$. Scale bar indicates 100 µm.

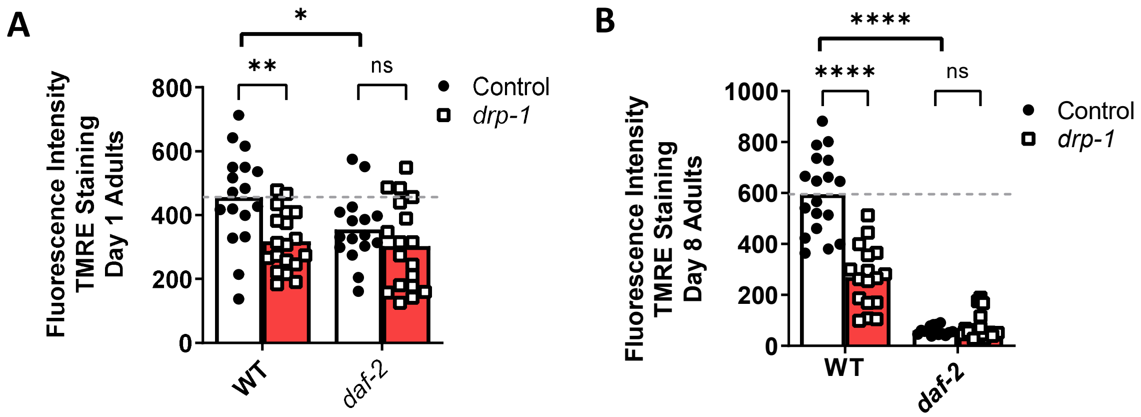

Fig. S9. Disruption of *drp-1* decreases mitochondrial membrane potential in wild-type worms but not *daf-2* mutants. Mitochondrial membrane potential as measured by whole-worm TMRE staining is decreased by disruption of *drp-1* in wild-type worms at day 1 (**A**) and day 8 (**B**) of adulthood. Disruption of *drp-1* does not further decrease mitochondrial membrane potential in *daf-2* mutants. Three biological replicates were performed. Statistical significance was assessed using a two-way ANOVA with Šidák's multiple comparisons test. Error bars indicate SEM. * $p<0.05$, **$p<0.01$, ****$p<0.0001$.

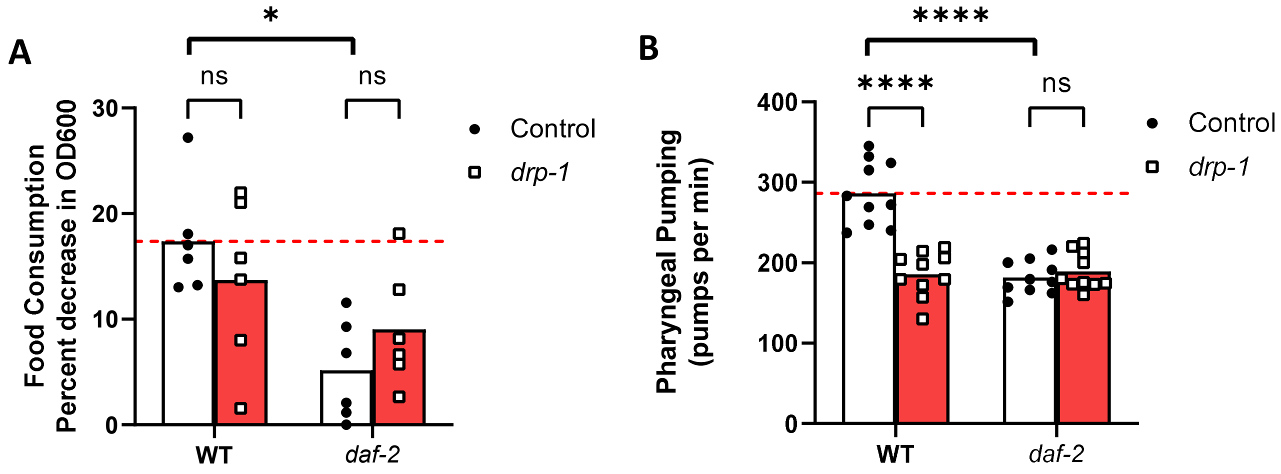

Fig. S10. Disruption of *drp-1* does not affect food consumption in *daf-2* worms. Food consumption was measured in two ways: by measuring the amount of OP50 bacteria consumed in liquid culture (**A**) or by measuring the rate of pharyngeal pumping (**B**). In both cases, *daf-2* worms exhibited decreased food consumption compared to wild-types animals. Disruption of *drp-1* did not significantly affect food consumption in *daf-2* worms but did decrease the pharyngeal pumping rate in wild-type animals. Statistical significance was assessed using a two-way ANOVA with Šidák's multiple comparisons test. ****$p<0.0001$.

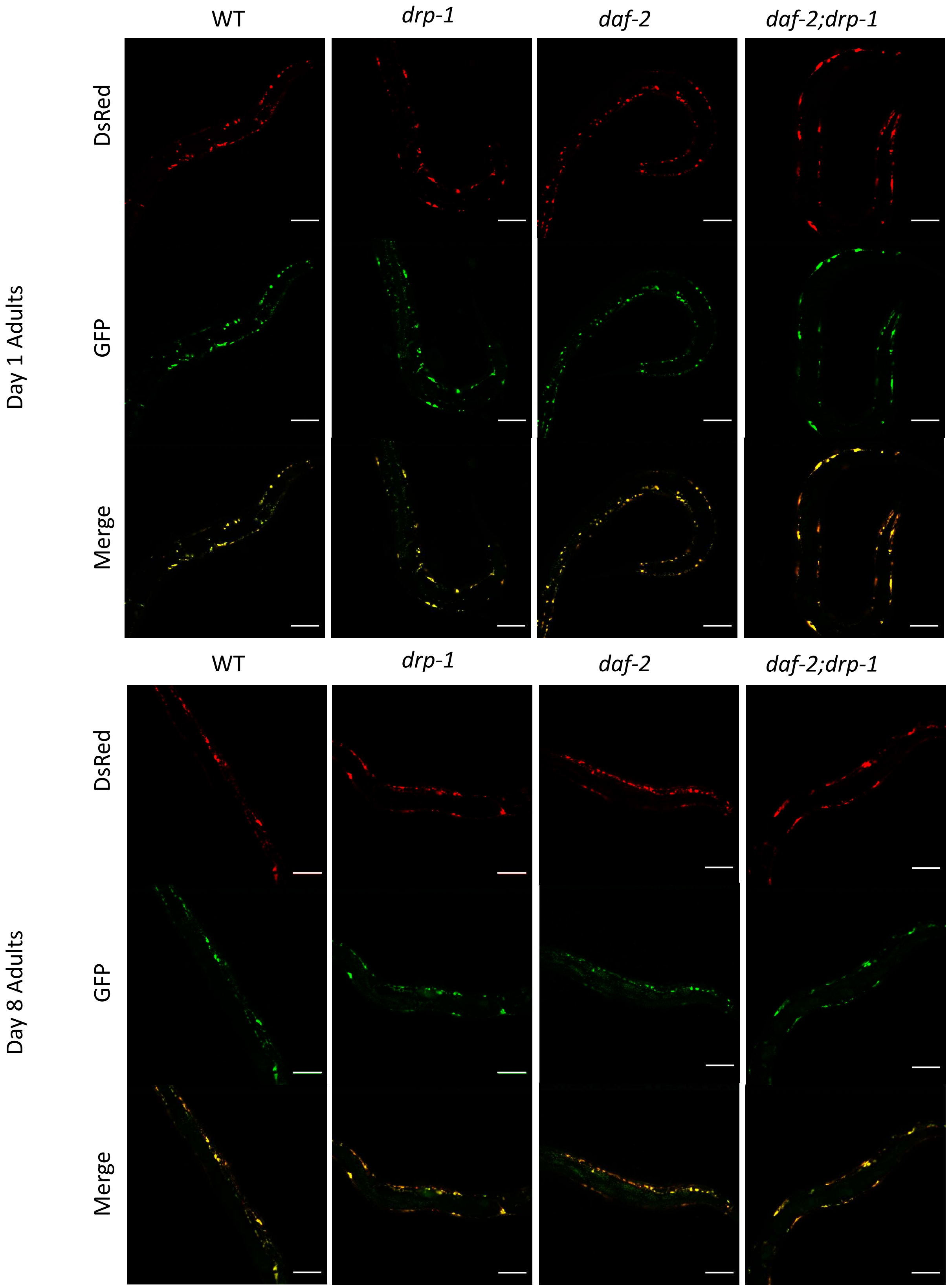

Fig. S11. Visualization of the mtRosella mitophagy reporter reveals that disruption of *drp-1* increases mitophagy in *daf-2* worms in day 1 young adults. Whole body images of animals expressing the mtRosella mitophagy reporter were obtained using dual-channel confocal microscopy. Mitophagy levels were determined by comparing the fluorescence intensity of pH-insensitive DsRed to pH-sensitive GFP. See **Fig. 8** for quantification of fluorescence. Representative images showing the compared intensity of each fluorophore were obtained by merging the two channels together. Scale bar represents 50 µm.

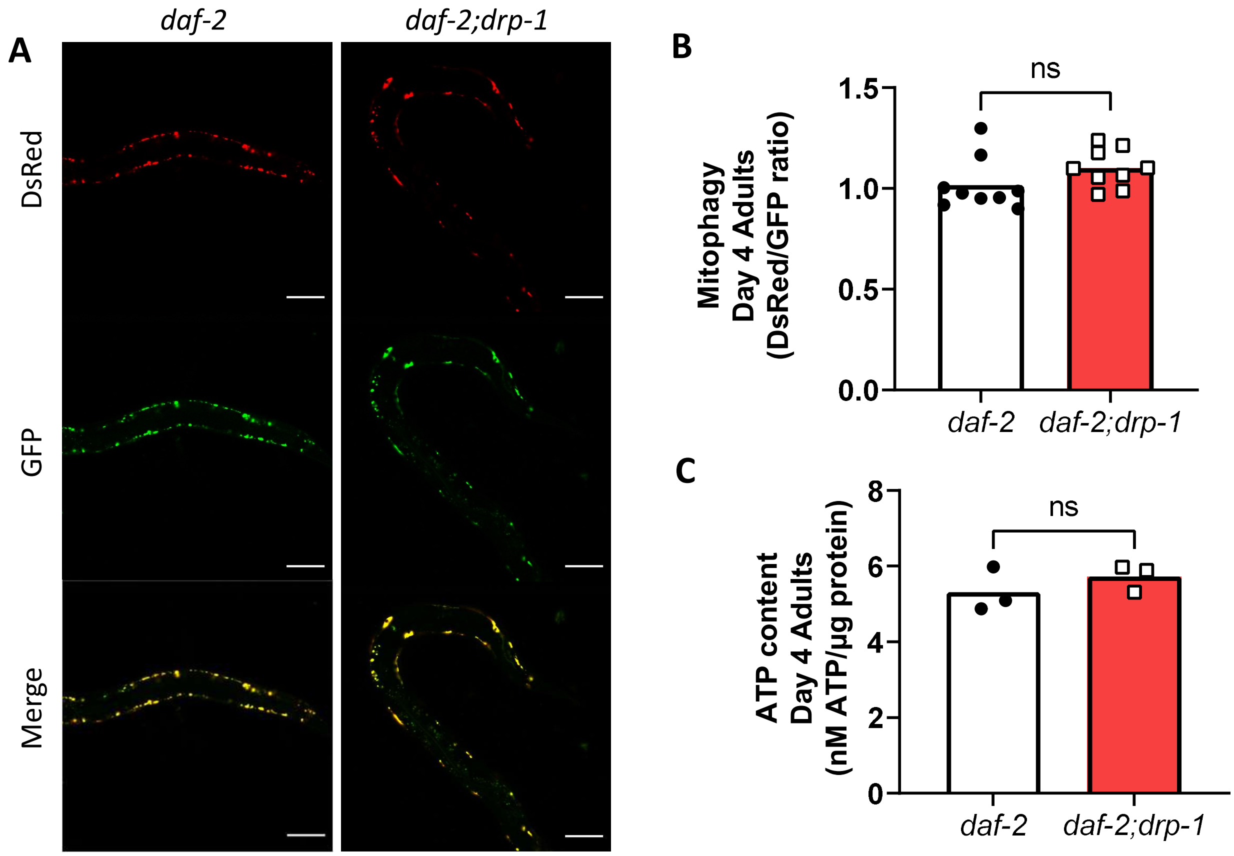

Fig. S12. The effect of *drp-1* deletion on mitophagy and ATP content in *daf-2* worms is lost by day 4 of adulthood. At day 4 of adulthood, mitophagy was quantified using the mtRosella reporter strain. Unlike at day 1 of adulthood, there was no difference between *daf-2* and *daf-2;drp-1* worms (**A,B**). Similarly, there was no difference in ATP levels between *daf-2* and *daf-2;drp-1* worms at day 4 of adulthood (**C**). Statistical significance was assessed using a student's t-test. Scale bar indicates 50 µm.

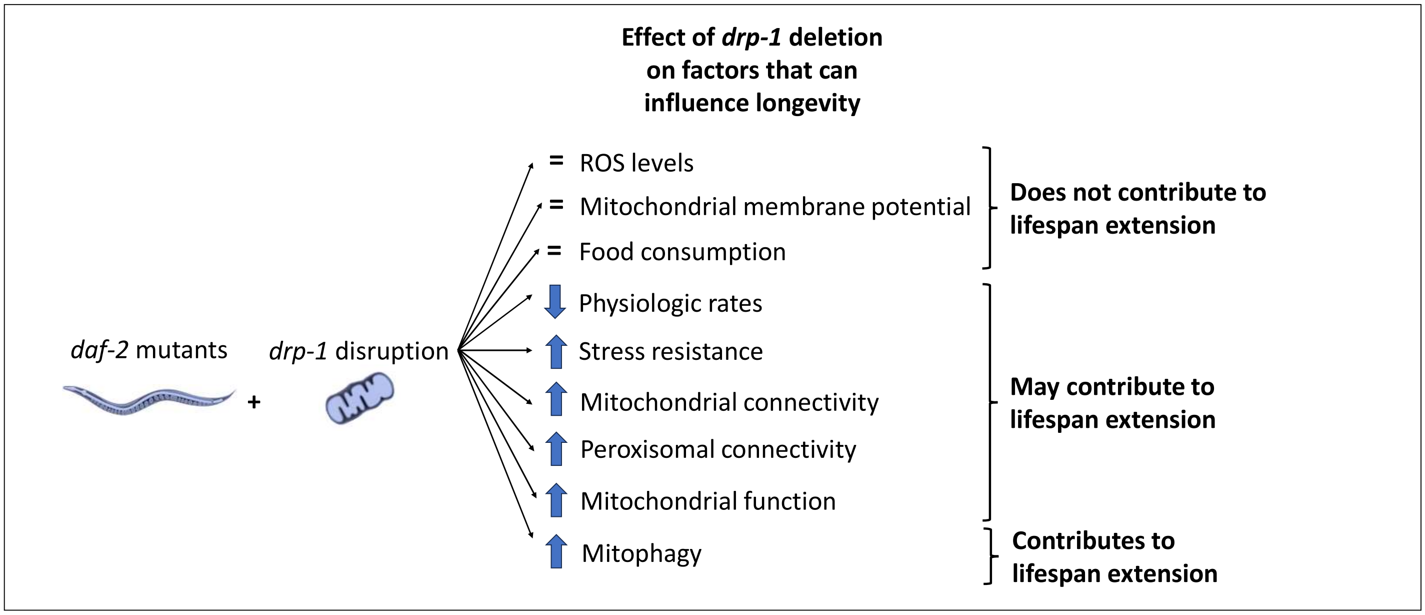

Figure S13. Factors contributing to extension of *daf-2* lifespan by disruption of *drp-1*. All of the factors tested have previously been shown to influence longevity. Factors that do not contribute to lifespan extension are those factors that were unaffected by *drp-1* disruption. Factors that may contribute to lifespan extension are those factors that are altered by *drp-1* and associated with longevity but that we were not able to directly test their contribution to lifespan extension. Factors that contribute to lifespan extension are those factors that are altered by *drp-1* and were shown to be required for *daf-2;drp-1* longevity. Down arrow indicates decreased, Up arrow indicates increased, = indicates unchanged.
