# Supplementary material for: Developmental disruption of the mitochondrial fission gene drp-1 extends the longevity of daf-2 insulin/IGF-1 receptor mutant
Source: GeroScience. 2024 Jul 19;47(1):877–902. doi: 10.1007/s11357-024-01276-z (PMC11872967; doi:10.1007/s11357-024-01276-z)
Supplement: Supplementary file 3 — Supplementary file3 (DOCX 23 KB) [file 11357_2024_1276_MOESM3_ESM.docx]

**Supplemental Tables

for**

**Developmental disruption of the mitochondrial fission gene *drp-1* extends the longevity of *daf-2* insulin/IGF-1 receptor mutant**

Annika Traa, Aura A. Tamez-González, Jeremy M. Van Raamsdonk

Table S2

Table S3

Table S4

Table S5

Note: Table S1 is a separate Excel file containing raw lifespan data

**Table S2. Effect of *drp-1* deletion in *daf-2* and wild-type background.**

| **Phenotype** | **Effect of *drp-1* deletion in**  ***daf-2* worms** | **Source** | **Effect of *drp-1* deletion in wild-type worms** | **Source** |
| --- | --- | --- | --- | --- |
| Lifespan | Increased | Figure 1 | Increased | Figure S1 |
| Thrashing rate – Day 1 | Decreased | Figure 1 | Decreased | Machiela et al. 2020, *FASEB J.* |
| Brood size | Decreased | Figure 1 | Decreased | Machiela et al. 2020, *FASEB J.* |
| Resistance to chronic oxidative stress | Increased | Figure 1 | Increased | Machiela et al. 2020, *FASEB J.* |
| Resistance to bacterial pathogens | Increased | Figure 1 | Not measured | N/A |
| Resistance to heat stress | Decreased | Figure 1 | Decreased | Machiela et al. 2020, *FASEB J.* |
| Resistance to acute oxidative stress | Decreased | Figure 1 | Increased | Machiela et al. 2020, *FASEB J.* |
| Resistance to osmotic stress | No effect | Figure 1 | Decreased | Machiela et al. 2020, *FASEB J.* |
| Resistance to anoxia | No effect | Figure 1 | Decreased | Machiela et al. 2020, *FASEB J.* |
| Number of mitochondria – Day 1 | Decreased | Figure 2 | No effect | Machiela et al. 2020, *FASEB J.* |
| Area of mitochondria – Day 1 | Increased | Figure 2 | No effect | Machiela et al. 2020, *FASEB J.* |
| Mitochondrial circularity – Day 1 | Increased | Figure 2 | No effect | Machiela et al. 2020, *FASEB J.* |
| Mitochondria Feret’s diameter – Day 1 | Decreased | Figure 2 | Not measured | Machiela et al. 2020, *FASEB J.* |
| Number of mitochondria – Day 8 | Decreased | Figure 2 | No effect | Machiela et al. 2020, *FASEB J.* |
| Area of mitochondria – Day 8 | Increased | Figure 2 | No effect | Machiela et al. 2020, *FASEB J.* |
| Mitochondrial circularity – Day 8 | Increased | Figure 2 | No effect | Machiela et al. 2020, *FASEB J.* |
| Mitochondria Feret’s diameter – Day 8 | No effect | Figure 2 | Not measured | N/A |
| Number of peroxisomes | Decreased | Figure 2 | Decreased | Figure S4 |
| Area of peroxisomes | No effect | Figure 2 | Increased | Figure S4 |
| Peroxisomal circularity | Decreased | Figure 2 | Decreased | Figure S4 |
| Peroxisome Feret’s diameter | Increased | Figure 2 | Increased | Figure S4 |
| Oxygen consumpt. – Day 1 | Increased | Figure 7 | No effect | Figure 7 |
| ATP levels – Day 1 | Increased | Figure 7 | No effect | Figure 7 |
| Mitophagy – Day 1 | Increased | Figure 8 | No effect | Figure 8 |
| ROS levels – Day 1 | No effect | Figure S8 | Increased | Figure S8 |
| ROS levels – Day 8 | No effect | Figure S8 | Increased | Figure S8 |
| Mitochondrial membrane potential – Day 1 | No effect | Figure S9 | Decreased | Figure S9 |
| Mitochondrial membrane potential – Day 8 | No effect | Figure S9 | Decreased | Figure S9 |
| Food consumption | No effect | Figure S10 | Decreased | Figure S10 |

**Table S3. Effect of disrupting mitochondrial fusion genes on mitochondrial morphology.** Results are from Machiela et al. 2020, *FASEB Journal*. NS=a non-significant trend was observed.

| **Phenotype** | **Effect of *fzo-1* deletion** | **Effect of *eat-3* deletion** |
| --- | --- | --- |
| Number of mitochondria – Day 1 | Increased | Increased |
| Area of mitochondria – Day 1 | Decreased | Decreased |
| Mitochondrial circularity – Day 1 | Increased | Increased |
| Number of mitochondria – Day 8 | Increased (NS) | Increased |
| Area of mitochondria – Day 8 | Decreased (NS) | Decreased |
| Mitochondrial circularity – Day 8 | Increased (NS) | Increased |

**Table S4. Effect of RNAi clones that decrease mitochondrial fragmentation in *daf-2* and wild-type background.**

| **RNAi clone** | **Effect on *daf-2* lifespan** | **Effect on wild-type lifespan** |
| --- | --- | --- |
| *drp-1* | Increased | No effect |
| *sdha-2* | Increased | Increased |
| C34B2.8 | Increased | No effect |
| K02F3.2 | Increased | No effect |
| T10F2.2 | Increased | Not measured |
| Y69F12A.b | Increased | Decreased |
| Y69F12A.c | Increased | Not measured |
| *timm17B.1* | Increased | No effect |
| C33A12.1 | Increased | Not measured |
| *cyp-35A1* | Increased | No effect |
| *pgp-3* | Increased | Increased |

**Table S5. Replication of phenotypes in *daf-2* worms**

| **Phenotype** | **Result from this paper** | **Previous results** |
| --- | --- | --- |
| ROS levels | Using DHE, we found ROS to be increased at Day 1 and unchanged at Day 8 in *daf-2* worms | Yang and Hekimi 2010 reported increased ROS in isolated mitochondria using MitoSox but no change with DCF in whole worms in *daf-2* worms  Brys et al. 2010 reported more H_2_O_2_ production in isolated mitochondria in *daf-2* worms  Zarse et al. 2012 reported decreased mitochondrial ROS using Mitotracker Red and decreased H_2_O_2_ production using Amplex Red in *daf-2* mutants, but transiently increased H_2_O_2_ production with *daf-2* RNAi |
| Mitochondrial membrane potential | Using TMRE, we found mitochondrial membrane potential to be decreased in *daf-2* worms | Brys et al. 2010 reported increased mitochondrial membrane potential in *daf-2* worms using DASPMI  Lemire et al. 2009 reported decreased mitochondrial membrane potential in *daf-2* worms using DiS-C3(3) |
| Oxygen consumption | Using Seahorse extracellular flux analyzer, we found oxygen consumption to be decreased in *daf-2* worms | Brys et al. 2010 reported increased oxygen consumption in *daf-2* worms  Zarse et al. 2012 reported increased respiration in *daf-2* worms |
| ATP levels | We found ATP levels to be increased in *daf-2* worms | Brys et al. 2010 reported increased ATP in *daf-2* worms  Zarse et al. 2012 reported increased ATP in *daf-2* worms |
| Mitophagy | Using mitoRosella reporter, we found mitophagy to be increased in *daf-2* worms | Palikaras et al. 2015 reported increased mitophagy in *daf-2* worms using LGG-1::GFP reporter to count autophagosomes |
| Food consumption | We found food consumption to be decreased in *daf-2* worms | Wu et al. 2019 reported decreased food consumption in *daf-2* worms |
